# Clinical application of a fully automated blood collection robot and its assessment of blood collection quality of anticoagulant specimens

**DOI:** 10.3389/fmed.2023.1251963

**Published:** 2023-09-05

**Authors:** Chong Wang, Meixiu Gu, Jie Zhu, Shuo Yang, Wenjia Tang, Zizhong Liu, Baishen Pan, Beili Wang, Wei Guo

**Affiliations:** ^1^Department of Laboratory Medicine, Zhongshan Hospital, Fudan University, Shanghai, China; ^2^Beijing Magicnurse Surgical Robot Technology Co. Ltd., Beijing, China; ^3^Department of Laboratory Medicine, Minhang Meilong Branch, Zhongshan Hospital, Fudan University, Shanghai, China; ^4^Department of Laboratory Medicine, Xiamen Branch, Zhongshan Hospital, Fudan University, Xiamen, China; ^5^Department of Laboratory Medicine, Wusong Branch, Zhongshan Hospital, Fudan University, Shanghai, China

**Keywords:** intelligent blood collection robot, blood collection, anticoagulation, patient experience, pre-analytical quality control

## Abstract

**Background and objectives:**

To investigate the application of intelligent puncture blood collection robots in anticoagulated blood specimens, the satisfaction of subjects with the two blood collection methods, and the feasibility of intelligent blood collection devices to replace manual blood collection methods in clinical work.

**Materials and methods:**

A total of 154 volunteers from Zhongshan Hospital Fudan University were recruited to compare the test results of anticoagulant blood samples between blood collection robot and manual blood collection, a questionnaire was used to inquire about the volunteers’ feelings about the two blood collection methods; the blood collection data of 6,255 patients willing to use the robot for blood collection were collected to analyze the success rate of blood collection.

**Results:**

The blood collection robot is superior to manual specimen collection in terms of volume and pain of specimen collection, and the puncture success rate is 94.3%. The anticoagulated blood specimens collected by the robot had 11 indexes statistically different from the results of manual blood collection, but the differences did not affect the clinical diagnosis and prognosis.

**Conclusion:**

The intelligent robotic blood collection is less painful and has better acceptance by patients, which can be used for clinical anticoagulated blood specimen collection.

## 1. Introduction

**Figure 1:**
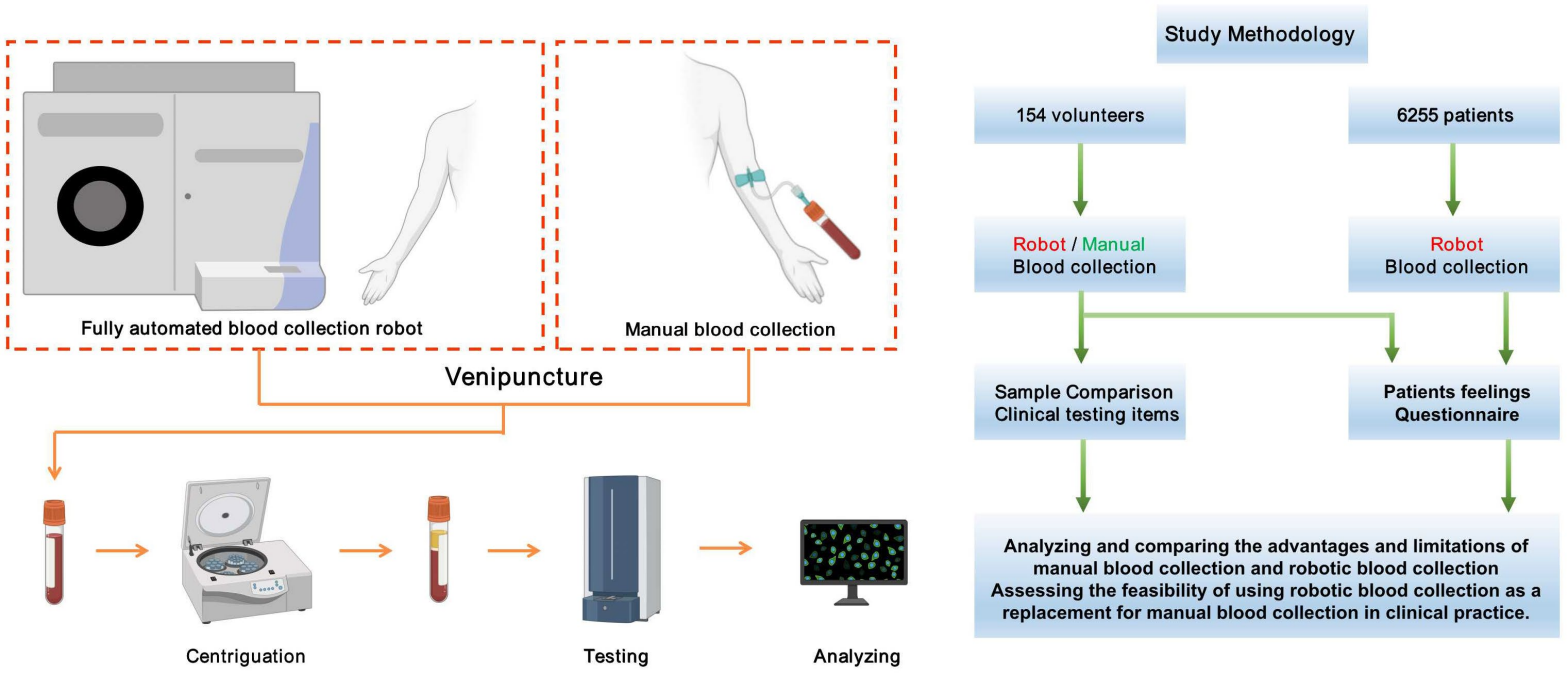
The schematic diagram of the experimental design for this study.

With the advancement of artificial intelligence devices in the healthcare domain, fully automatic intelligent blood collection devices are finding increasing application in clinical settings. This study aims to explore the novelty of the MagicNurse Intelligent Venipuncture & Blood Collection Robot by evaluating the quality of specimens it collects, comparing the time consumption with conventional manual blood collection, and assessing subjects’ perceptions of the two different blood collection methods. Specifically, we will discuss the application of this intelligent robot in handling anticoagulated blood specimens, measure the satisfaction levels of the subjects with both blood collection approaches, and explore the feasibility of replacing manual methods with intelligent blood collection devices in clinical practice. The experimental design scheme for this study is presented in Figure 1.

## 2. Materials and methods

### 2.1. Study subjects

A total of 154 volunteers were recruited from Zhongshan Hospital, Fudan University. Each participant used the same brand and batch of vacuum blood collection tubes to collect a total of 4 tubes of blood – one EDTA-K2 anticoagulated blood, one citrate anticoagulated blood, and two serum procoagulant blood samples. The blood collection was performed using both the intelligent blood collection robot and manual methods with a time interval of 10 min between each collection. Throughout the blood collection process, we measured the time consumption for each blood draw using a stopwatch, and also recorded the duration of arm fixation ring action during blood collection by the robot. After the blood collection, the patients were given a questionnaire to express their feedback and feelings about the two blood collection methods. The EDTA-K_2_ anticoagulant specimens underwent the complete blood count (CBC) test within half an hour after blood collection; while the citrate anticoagulant specimens were centrifuged within half an hour after blood collection, and prothrombin time (PT), international normalized ratio (INR), thrombin time (TT), activated partial thromboplastin time (APTT), fibrinogen (Fib), D-dimer (D-D), and fibrin(−ogen) degradation products product (FDP) assays were analyzed within one hour.

From September 2021 to August 2022, 6,255 patients who were willing to use intelligent robots for specimen collection in the daily blood collection work of Zhongshan Hospital Fudan University were enrolled, and the overall success rate and puncture success rate of intelligent robots for blood collection were evaluated, and the reasons for blood collection failure were analyzed.

The blood collection robot had been approved with the NMPA (National Medical Products Administration) Class III medical device registration certificate, and had obtained the production license in 2020. The hospital ethics committee approved this study. In the study, all participants signed informed consent forms.

### 2.2. Instruments reagents

MagicNurse Intelligent Venipuncture & Blood Collection Robot and supporting consumables (MagicNurse Beijing Ltd.); Vacuum blood collection tube (Xinle Medical); Sysmex XN-20 automatic hematology analyzer and supporting reagents (Sysmex); Sysmex CN-6000 automatic coagulation analyzer (Sysmex).

### 2.3. Blood collection methods

The preferred site for manual specimen collection is the antecubital vein in the arm. A vacuum blood collection tube is utilized during the process to create a pressure difference, allowing the blood to naturally flow into the tube. Following successful collection, the sample is manually mixed.

In contrast, the intelligent robot primarily targets the median vein for blood collection. Utilizing artificial intelligence machine vision technology and image navigation control technology, blood collection robots can accurately identify, locate, and collect blood vessels. To establish this technology, a large vascular imaging database was created based on vascular images collected from diverse domestic and foreign populations in the early stages. Through this database, a big data algorithm for different population vascular images was obtained.

**Figure 2:**
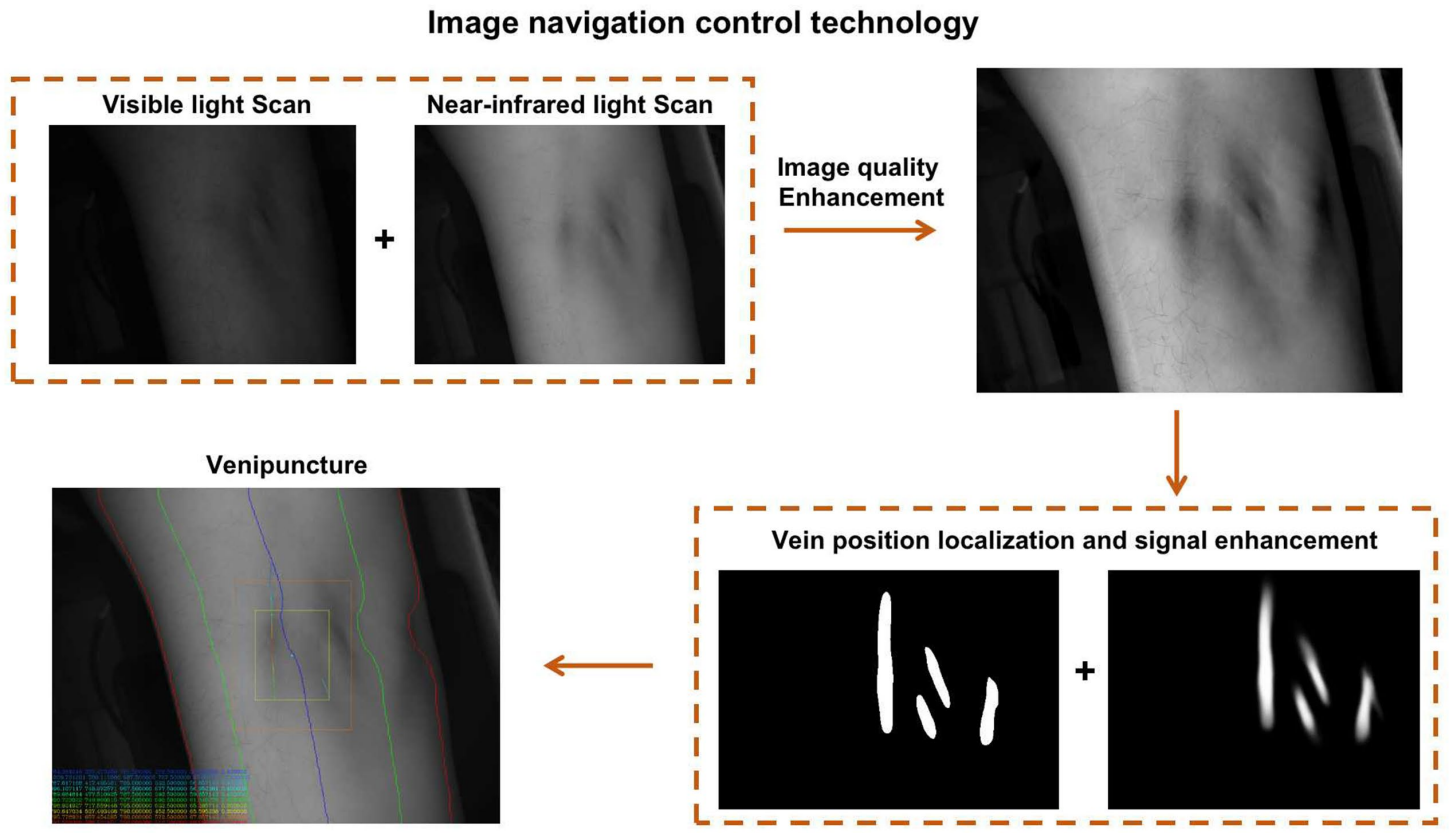
The schematic diagram illustrating the working principle of the image navigation control technology in the blood collection robot.

Additionally, the image navigation control technology enables the robot to use near-infrared and visible light to accurately identify blood vessels, puncture points, puncture angles, puncture paths, and forces, catering to venous vessels with varying orientations. This precise planning offers technical support for the precise puncture of blood collection needles (Figure 2).

During the blood collection, the intelligent robot first adjusts the pressure inside and outside the collection tube to maintain consistency. It simultaneously inserts both the blood collection needle and the negative pressure suction needle into the vein. A constant external pressure is applied to extract gas from the collection tube, creating the pressure difference for blood collection. The intelligent blood collection system sets corresponding blood collection volumes based on different types of vacuum blood collection tubes. The system automatically accounts for the volume of the butterfly needle system’s tubing during the first specimen collection, avoiding inaccuracies caused by “dead space” volume.

After each specimen collection, the internal robot manipulator performs six standardized mixing actions on the blood collection tube (gentle inversion of the specimen by 180° and resetting as one mixing action) and places the tube in the specimen collection box.

### 2.4. Assessment of pre-test impact factors for both blood collection methods

The assessment of the pre-test impact factors for the two blood collection methods encompassed several aspects. These factors included the time required for blood collection, the sequence of collecting different blood collection containers during specimen collection, the adequacy of the blood collection volume, the accuracy of label identification, the alignment of test items with the specimen type, and the properties of the specimens

### 2.5. Evaluation of volunteer subjects’ satisfaction with the two blood collection methods

Statistical analysis was performed by using questionnaires on the painfulness of the two blood collection methods and the willingness of the volunteer subjects to collect blood from the intelligent robot in the future after they finished blood collection. (The online questionnaire website is: https://www.wjx.cn/vm/tVZlkvR.aspx)

### 2.6. Evaluation of the success rate of blood collection by the intelligent blood collection robot

Data analysis was conducted on 6,255 patients who willingly participated in the daily use of the intelligent robotic specimen collection. The success rate of robotic blood collection was calculated based on this dataset. All specimens were collected in a single blood collection process, ensuring that the volume of each blood sample met the specified extraction requirement. Moreover, anticoagulated samples were carefully examined to ensure they were free from clotting. Meeting these conditions qualified the blood collection process as successful.

### 2.7. Comparison of anticoagulation test results between the two blood collection methods

EDTA-K2 anticoagulated blood and citrate anticoagulated blood collected using the two blood collection methods were subjected to CBC tests and coagulation tests. The test results were categorized into the robotic and manual groups, and the differences between the two groups of data were analyzed and compared.

### 2.8. Statistical methods

The SPAA 17.0 software was used for statistical analysis of the data, and t-test and ANOVA tests were used for comparison of the test results, with *a* = 0.05 as the test level and *p* < 0.05 as a statistically significant difference. Among them, for data with non-normal distribution, the rank sum test was used for statistics.

## 3. Results

### 3.1. Assessment of pre-test impact factors for both blood collection methods

The blood collection sequence for both manual blood collection and intelligent robotic blood collection followed this order: citrate anticoagulation tube → serum pro-coagulation tube → EDTA-K2 anticoagulation tube. Throughout the study, no misidentification of specimen uniqueness or misuse of vacuum blood collection tubes was observed. The pre-test impact factors of the two blood collection methods are displayed in Table 1, which indicated that the robotic blood collection time was significantly longer than the manual blood collection time, with a statistically significant difference (*p* < 0.05). Besides, a total of 60 cases experienced insufficient specimen volume, with 12 cases related to anticoagulated blood samples. In contrast, no instances of insufficient specimen volume were observed in the blood samples collected using the robotic blood collection method, when compared to manual collection.

**Table 1:** Statistics of pre-analytical influencing factors between manual blood collection and intelligent robotic blood collection.

		Time of blood collection(s)	Robot *vs* manual (p)	Duration of tourniquet action (s)	Robot *vs* manual (p)	Duration of arm immobilization belt action (s)	No. of specimen column	No. of tube column	No. of tubes with low blood collection volume		No. of cases of clots visible
									Anticoagulation tube	Serum Tube	
Robotic blood collection		204.9 ± 1.9	*p* < 0.001	47.8 ± 0.7	*p = 0.893*	179.9 ± 2.1	154	616	0	0	0
Manual blood collection	Nurse A*	48.9 ± 3.3		43.2 ± 3.4		/	64	256	4	19	0
	Nurse B*	49.8 ± 3.4		44.2 ± 4.8		/	64	256	7	25	0
	Nurse C*	64.7 ± 3.1		57.7 ± 3.0		/	26	104	1	4	0
	Total of manual method	51.9 ± 7.1		46.1 ± 6.6		/	154	616	12	48	0

*A, B, and C represent 3 nurses with different blood collection skills, Nurse A has the best blood collection skills, Nurse B has medium blood collection skills and Nurse C has the worst blood collection skills.

### 3.2. Evaluation of volunteer subjects’ satisfaction with the two blood collection methods

The statistical data from 154 volunteers regarding the pain experienced during the two blood collection methods and their willingness to use robots for blood collection are presented in Table 2. The experimental findings revealed that compared to manual blood collection, using the robotic blood collection method resulted in a milder pain sensation for patients. Furthermore, out of the 154 patients, 108 patients expressed their willingness to use the robotic blood collection method for future blood specimen collection.

**Table 2:** The result of questionnaires for 154 volunteers.

	No. of volunteers	Age	Robotic blood collection		Manual blood collection		Which is more painful			No. of willingness to use robotic blood collection
			Left Hand	Right Hand	Left Hand	Right Hand	Robot	Manual	Same pain	
Male	70	28 ~ 81 (50.57 ± 10.02)	31	38	36	34	5	36	29	47
Female	84	23 ~ 77 [50 (40.1, 57)]	34	50	52	32	12	54	18	61
Total	154	23 ~ 81 [51 (43, 57)]	65	88	88	66	17	90	47	108

*For data with normal age distribution, the mean ± standard deviation was used; for data with non-normal distribution, the median and quartiles were used.

### 3.3. Evaluation of the success rate of blood collection by the intelligent blood collection robot

The statistical analysis of the 6,255 cases, who expressed a willingness to use the intelligent robot for specimen collection in their daily blood collection, revealed that 164 patients were excluded from the scope of robotic blood collection due to the presence of tattoos on their arms or scars on the skin of their blood vessels. Additionally, 75 patients voluntarily opted out of robotic blood collection for personal reasons. Among the 6,016 patients who actually underwent robotic blood collection, the instrument achieved a puncture success rate of 94.3% (Table 3).

**Table 3:** Statistics of 6,255 patients who were willing to use robotic blood collection.

No. of patients willing to robotic blood collection	No. of patients beyond the scope of use	No. of patients who voluntarily abandoned robotic blood collection	No. of patients underwent robotic blood collection	No. of patients who were rejected by the robot	No. of patients actually punctured by the robot	No. of successful robotic blood collection	Success rate of puncture blood collection
6,255	164	75	6,016	50	5,966	5,628	94.3%

### 3.4. Comparison of anticoagulation test results between the two blood collection methods

The results of the relevant indexes of the anticoagulated blood specimens collected under the two blood collection methods are shown in Tables 4, 5. The results of the five items of red blood cell count, hemoglobin concentration, hematocrit, red blood cell volume distribution width SD, and mean platelet volume in the two groups of EDTA-K_2_ anticoagulated blood specimens were significantly different (*p* < 0.05); the results of the two groups of citrate anticoagulated blood specimens of PT, INR, TT, APTT, Fib, and D-D were significantly different (*p* < 0.05).

**Table 4:** Differences in the results of EDTA-K2 anticoagulated blood specimens between the two blood collection methods (*N* = 154).

	Unit	Robotic group	Manual group	t/Z	*p*
Red blood cell count^a^	×10^12^/L	4.763 ± 0.480	4.823 ± 0.486	−6.636	0.000
Hemoglobin^a^	g/L	142.275 ± 16.747	144.065 ± 16.819	−7.364	0.000
Hematocrit^a^	%	43.084 ± 4.308	43.666 ± 4.349	−7.171	0.000
Mean corpuscular volume^b^	fL	91.4 (89.0, 93.2)	91.6 (89.1, 93.6)	1.834	0.067
Mean corpuscular hemoglobin^b^	pg	30.2 (29.4, 30.9)	30.4 (29.4, 31.1)	0.572	0.567
Mean corpuscular hemoglobin concentration^a^	g/L	329.758 ± 11.658	329.471 ± 12.029	0.712	0.478
Platelet count^a^	×10^9^/L	241.327 ± 57.390	238.928 ± 56.620	1.788	0.076
White blood cell count^b^	×10^9^/L	5.69 (4.55, 6.83)	5.70 (4.60, 6.67)	−0.836	0.403
Neutrophilic granulocyte^a^	%	57.582 ± 8.432	57.849 ± 8.422	−1.636	0.104
Lymphocyte^a^	%	32.733 ± 7.992	32.496 ± 7.834	1.558	0.121
Monocyte^a^	%	6.839 ± 1.504	6.837 ± 1.489	0.053	0.958
Eosinophilic granulocyte^b^	%	1.8 (0.9, 3.1)	1.8 (0.9, 3.0)	0.897	0.370
Basophilic granulocyte^b^	%	0.4 (0.3, 0.6)	0.4 (0.2, 0.6)	0.062	0.950
Red blood cell volume distribution width-CV^b^	%	12.3 (11.9, 12.7)	12.3 (11.9, 12.7)	0.128	0.898
Red blood cell volume distribution width-SD^b^	fL	41.2 (39.4, 42.9)	41.1 (39.5, 43.0)	2.285	0.022
Mean platelet volume^b^	fL	10.0 (9.4, 10.6)	10.0 (9.4, 10.6)	2.815	0.005
Plateletocrita	%	0.242 ± 0.052	0.286 ± 0.056	−0.95	0.344
Platelet large cell ratio^b^	%	24.9 (20.2, 29.6)	25.0 (20.0, 30.0)	1.679	0.093
Platelet volume distribution width^b^	%	11.1 (10.1, 12.5)	11.1 (10.2, 12.4)	0.706	0.480

a Normally distributed data, using paired *t*-test statistics, and statistical data are expressed as mean ± standard deviation.

b Non-normally distributed data, using rank sum test statistics, and statistical data are expressed by median and quartile.

**Table 5:** Differences in the results of citrate anticoagulated blood specimens between two blood collection methods (*N* = 154).

	Unit	Robotic group	Manual group	t/Z	*p*
PT^b^	s	11.1 (10.7, 11.5)	11.1 (10.7, 11.4)	2.145	0.032
INR^a^	/	0.954 ± 0.059	0.952 ± 0.056	2.081	0.039
TT^b^	s	17.8 (17.4, 18.125)	18.2 (17.7, 18.6)	7.881	0.000
APTT^a^	s	26.024 ± 1.761	26.220 ± 1.822	−5.196	0.000
Fib^b^	mg/dL	298.0 (260.0, 331.23)	288.0 (250.0, 326.0)	3.944	0.000
D-Dimer^b^	mg/L	0.21 (0.15, 0.39)	0.21 (0.15, 0.38)	2.334	0.020
FDP^b^	μg/mL	0.805 (0.47, 1.1475)	0.850 (0.45, 1.1475)	0.223	0.823

a Normally distributed data, using paired *t*-test statistics, and statistical data are expressed as mean ± standard deviation.

b Non-normally distributed data, using rank sum test statistics, and statistical data are expressed by median and quartile.

## 4. Discussion

Comprehensive quality control in medical testing laboratories involves three critical phases: pre-analytical quality control, analytical quality control, and post-analytical quality control. Given the high rate of unqualified specimens attributed to pre-analytical errors, ranging from 46.0 to 68.2% (1, 2), pre-analytical quality management has emerged as a focal point in the overall quality control process.

In this study, 154 volunteers underwent two blood collection methods within a short period, utilizing the same brand, type, and lot number of blood specimen vacuum collection tubes for both groups. To ensure the consistency of all influencing factors during the pre-analysis and analysis stages, except for the different blood collection methods, the specimen pre-treatment methods and testing instruments were kept identical, ensuring the reliability of the study results.

Concerning the blood collection methods, in manual blood collection, blood flow automatically stops after the vacuum in the blood collection tube is depleted (3). Consequently, variations in blood collection tube quality and differences in manual collection may lead to varying degrees of insufficient specimen volume.

The duration of manual collection varied depending on the proficiency of the staff, the number of blood collection tubes, and the vascular condition of the patients. In this study, it took 51.9 ± 7.1 s for the manual collection of 4 tubes of specimens from the beginning of binding the tourniquet to the end of the blood collection, and the action time of the tourniquet was 46.1 ± 6.6 s, which is in line with the suggestion in the relevant national standards that the use time of tourniquets should not exceed 1 min (3, 4). The entire process of robot blood collection which is set by the computer system involves arm fixation, precise identification and positioning of blood vessels and veins, grasping of the blood collection needle, and blood collection. Table 1 shows the total blood collection time of the robot (204.9 ± 1.9 s) was significantly higher than that of the manual group (51.9 ± 7.1 s). Although the tourniquet lasted less than 1 min, the arm fixation belt also exerts a certain pressure on blood vessels when the robot collects blood, and the duration is longer, which may have an impact on the detection results (5, 6). The blood collection settings need further improvement.

The results of the questionnaire survey among 154 volunteers showed that 90 volunteers thought that the robot blood collection was less painful while 17 volunteers thought that the manual blood collection was less painful. Pain is a subjective sensation produced by pain receptors on nerve endings. The fact that the pain of robot blood collection is lighter depends on two aspects. On the one hand, it depends on the multiple positioning of the skin puncture point and the precise needle insertion before the blood collection, which can puncture the skin and blood vessels in place at one time and shorten the process of the mechanical stealth, and the speed is fast, thus, the damage to the skin and blood vessels was reduced, and the pain released by the pain receptors was reduced. Additionally, the reduced pain sensation may also be due to an excess of adrenaline secretion during robot-assisted blood collection for some volunteers. In the questionnaire survey, 108 volunteers were willing to try using the robot to collect blood again, mainly because the pain caused by robot blood collection is lighter than that of manual blood collection. During peak outpatient blood collection, robot blood collection does not require queuing or the waiting time is shorter than that of manual blood collection, resulting in a better patient experience. Besides, 46 volunteers were asked why they did not want to use the robot, 17 volunteers said the robot felt more painful, and another 29 volunteers believed that the robot blood collection time was too long and did not want to use it again. The improvement of the robot blood collection process and speed may further improve patients’ blood collection experience and increase patients’ willingness to use the robot for blood collection.

During the process of automated robotic blood collection, the whole operation is carried out in an enclosed space inside the instrument. The whole process of collecting blood can be viewed through real-time monitoring equipment, which is a good choice for patients suffering from fainting needles or fainting blood. However, some patients refuse to try robot blood collection because of their weak acceptance of new things, the inability to communicate with the staff compared with manual blood collection, and the fear of blood collection failure causing unnecessary follow-up troubles. In this study, the puncture success rate of robot collection was higher than that of the general out-patient population (7). Although there are certain differences in the population selection for this comparison, from a certain perspective, it also reflects the higher success rate of the blood collection robot in puncture. The success rate of one puncture is low due to the inexperienced blood collection skills of some staff members, and repeated punctures can increase the pain of patients and cause medical disputes. Automated robotic blood collection equipment has a high puncture success rate and can be promoted as a supplement to traditional blood collection methods. In addition, we conducted a causal analysis on 50 patients who were refused blood sampling by robots. One was that the blood vessels of patients could not be identified. During robotic blood collection, the patient needs to hold the instrument’s push-pull lever and move inward to fix the upper end of the arm to prevent significant arm movement. Due to the limited internal space of the instrument, the moving distance of the push-pull rod movement is fixed. Therefore, for patients with long or short arms, the robot could not identify the blood vessels. Second, it rejected a small number of patients with deep and curved vessels because of the high risk of vascular puncture failure. Of the 388 patients who failed to collect blood from the robot, 97 patients had punctured veins located on both sides of the arm, and the robot arm holding needle failed to collect blood because of the movement angle. 86 patients’ arms were displaced after vein positioning due to nervousness and arm swings, resulting in the robot failing to collect blood. For the remaining 155 patients whose blood vessels were in normal position but the puncture failed, the main reason was that the blood vessels were thinner, and repeated searching for vein and vein orientation led to the time-out of blood collection. The discovery of these problems will help us further optimize the settings and procedures of the blood collection robot to serve more patients.

The pre-analytical factors affecting the test results mainly involved specimen collection, specimen transportation, specimen stability, and specimen handling (8, 9). In this study, the specimens were uniformly transported and processed immediately after collection, so the differences in the results of anticoagulant blood specimens in this study mainly came from the specimen collection process. After analysis and discussion, we believe that the main reasons for the differences in the results of anticoagulant blood specimens between the two blood collection methods are: (1) Differences in blood collection needles: studies have shown that different sizes of blood collection needle will have a certain impact on platelet and coagulation test results (10). In this study, the size of a butterfly-wing needle for robot blood collection is 0.8 × 26 TWLB (21G) (11, 12), and the size of a straight needle for manual blood collection is 21G. The size of the two needles is equal, and there is no difference in the diameter of skin and vascular puncture points, the blood cells flow through the blood collection needle with the same aperture size, but as the robot uses the butterfly-wing needle to collect blood, there is no anti-coagulant added in the hose of the butterfly-wing needle system, which may cause differences in the test results, (2) The difference in the time of vascular compression: the time of vascular compression has an influence on the results of CBC and coagulation tests. Excessive compression time will lead to an increase in red blood cell count and hemoglobin concentration, the shortening of PT, TT, and APTT times, and an increase in fibrinogen concentration (5, 6). In the present study, although the tourniquet duration was less than the standard 1 min for both methods of blood collection, the results may have been influenced by the use of an arm-fixing ring when the robot was collecting blood, with its force pressing on the upper end of the arm for a duration of more than 1 min, (3) The influence of the dose and concentration of anticoagulant in the specimen collection tube on the result: for the coagulation tests, the accuracy of the anticoagulant dose and concentration is directly related to the correctness of the test results. Under normal circumstances, 0.3 mL of 0.109 mol/L citric anticoagulant should be mixed with blood in a 1:9 ratio (3, 4, 13). If the concentration or the volume of anticoagulants increases at the time of sampling, there will still be an overdose of anticoagulants in the case of full blood collection, resulting in prolonged PT, TT, and APTT time and decreased fibrinogen concentration (14). If the required volume of blood is not taken, it will aggravate the mismatch between the anticoagulants and the blood and make the test results more abnormal (15), and (4) The impact of the instantaneous negative pressure increase on the test results: the robot is set to collect blood at a constant pressure, while when blood is collected manually, the instantaneous negative pressure in the vacuum tube is large at the initial stage of blood collection, the blood flow speed is too fast, and the cells collide with each other (16), which may cause the increase of blood cell components flowing into the vacuum blood collection tube, and the change of the shape and size of the cells due to the collision with each other, which is consistent with the different results of the relevant data in Tables 4, 5) The influence of sample mixing method after specimen collection: the correct mixing method after specimen collection is very important for the test results, especially for the coagulation tests. Several studies have shown that clots in specimens are the main cause of substandard specimens in anticoagulated blood specimens (17–19). Visible clots can be identified by examining the specimen characteristics, but for tiny clots that cannot be identified by the naked eye due to insufficient or untimely mixing of blood and anticoagulants or unsuccessful blood collection, significant differences in the detection results may occur. In this study, the samples were mixed 6 times after the blood collection by the robot (the specimen was gently inverted 180° and reset for 1 Time), while manual specimen mixing was difficult to unify, which may be one of the reasons for the difference in the test results between the two blood collection methods. Although some results of anticoagulated blood specimens obtained by the two blood collection methods were significantly different, the percentages of the difference between the two did not exceed the relevant health industry standards and the local clinical laboratory quality management standards issued by the National Health Commission of the PRC and Shanghai Center for Clinical Laboratory (20, 21).

In this study, we compared the quality of samples collected between automated robot blood collection and manual blood collection. Statistical data showed that the automated robot blood collection system exhibited high standardization in the blood collection process, with higher accuracy in specimen volume and mixing quality compared to manual collection. The difference in test results of anticoagulated blood specimens collected by the two methods was clinically acceptable. Additionally, the automated robot blood collection provided a positive patient experience, especially for those with a history of fainting due to needles and blood. Moreover, it reduced the occupational exposure of medical staff to needle-stick injuries and contact infections. However, some deficiencies were noted in the automated blood collection robot, such as extended blood collection time causing patient anxiety and limitations in blood collection from both arms. Further research will focus on biochemical and immunobiological indicators to observe the clinical applicability of specimens collected by automated blood collection robots.

## Data availability statement

The raw data supporting the conclusions of this article will be made available by the authors, without undue reservation.

## Ethics statement

The studies involving humans were approved by Zhongshan hospital ethics committee. The studies were conducted in accordance with the local legislation and institutional requirements. The participants provided their written informed consent to participate in this study.

## Author contributions

WG and BW had full access to all of the data in the study and take responsibility for the integrity of the data and the accuracy of the data analysis. WG, BW, BP, CW, MG, JZ, SY, WT, and ZL: study concept and design. CW and MG: acquisition of data. JZ, CW, and ZL: analysis and interpretation of data. CW, JZ, and SY: drafting the manuscript. WG and BW: critical revision of the manuscript for import intellectual content. JZ and CW: statistical analysis. WG, BW, and JZ: obtained funding. ZL, WT, and CW: administrative, technical, and material support. CW, MG, WT, JZ, and SY: study supervision. All authors contributed to the article and approved the submitted version.

## Funding

This research was funded by the National Natural Science Foundation of China (82202608, 82172348, 81902139, and 81972000), the Constructing Project of Clinical Key Disciplines in Shanghai (shslczdzk03302), the Specialized Fund for the Clinical Researches of Zhongshan Hospital, Fudan University (2020ZSLC54), the Key Medical and Health Projects of Xiamen (YDZX20193502000002), Shanghai Baoshan Medical Key Specialty (BSZK2023A18).

## Conflict of interest

ZL was employed by Beijing Magicnurse Surgical Robot Technology Co. Ltd.

The remaining authors declare that the research was conducted in the absence of any commercial or financial relationships that could be construed as a potential conflict of interest.

## Publisher’s note

All claims expressed in this article are solely those of the authors and do not necessarily represent those of their affiliated organizations, or those of the publisher, the editors and the reviewers. Any product that may be evaluated in this article, or claim that may be made by its manufacturer, is not guaranteed or endorsed by the publisher.

## References

[1] Abdollahi A Saffar H Saffar H. Types and frequency of errors during different phases of testing at a clinical medical Laboratory of a Teaching Hospital in Tehran. Iran N Am J Med Sci. (2014) 6:224–8. doi: 10.4103/1947-2714.132941, PMID: 24926448 PMC4049056

[2] Plebani M. Errors in clinical laboratories or errors in laboratory medicine? Clin Chem Lab Med. (2006) 44:750–9. doi: 10.1515/CCLM.2006.123, PMID: 16729864

[3] Pan BS Guo W Wang BL Xu JM Hao XK Zhao J. WS/T 661–2020, guidelines of venous blood specimen collection. (2020). (in Chinese).

[4] Peng MT Gu XL Shi LF Li CB Shen ZY. WS/T 359–2011, collection and processing of blood specimens for testing plasmabased coagulation assays. (2011). (in Chinese).

[5] Connes P Uyuklu M Tripette J Boucher JH Beltan E Chalabi T. Sampling time after tourniquet removal affects erythrocyte deformability and aggregation measurements. Clin Hemorheol Microcirc. (2009) 41:9–15. doi: 10.3233/CH-2009-1146, PMID: 19136737

[6] Lippi G Salvagno GL Montagnana M Guidi GC. Short-term venous stasis influences routine coagulation testing. Blood Coagul Fibrinolysis. (2005) 16:453–8. doi: 10.1097/01.mbc.0000178828.59866.03, PMID: 16093738

[7] Mao Y. Nursing measures to improve the success rate of venous blood sampling and puncture for outpatient nurses. Med. Forum. (2017) 21:37056. (in Chinese)

[8] Gosselin RC Marlar RA. Preanalytical variables in coagulation testing: setting the stage for accurate results. Semin Thromb Hemost. (2019) 45:433–48. doi: 10.1055/s-0039-1692700, PMID: 31291676

[9] Kayadibi H Acar IA Cam S. Stability of complete blood count parameters depends on the storage temperature, storage time, transport position and selected stability criterion. Scand J Clin Lab Invest. (2020) 80:470–8. doi: 10.1080/00365513.2020.1783570, PMID: 32597228

[10] Lippi G Salvagno GL Montagnana M Poli G Guidi GC. Influence of the needle bore size on platelet count and routine coagulation testing. Blood Coagul Fibrinolysis. (2006) 17:557–61. doi: 10.1097/01.mbc.0000245300.10387.ca, PMID: 16988551

[11] Zhang HH Song JZ Wu P Jia YF. GB 18671–2009, intravenous needles for single use. (2009). (in Chinese).

[12] Li WY Liu XL Hua SH Zhang Q Huang BH Tian XL. YY/T 0296–2022, hypodermic needles for single use—Colour coding for identification. (2022). (in Chinese).

[13] Shang H Wang YS Shen ZY. Fourth edition of National Guide to clinical laboratory procedures. (People’s Med Publishing House Press). (2015) 160–74. (in Chinese)

[14] Edward W. Clinical laboratory diagnostics: Use and assessment of clinical laboratory results. Lothar Thomas. Frankfurt/Main, Germany: TH-Books Verlagsgeselschaft (1998). 1727 p ISBN 3-9805215-4-0.

[15] Gros N Klobučar T Gaber K. Accuracy of citrate anticoagulant amount, volume, and concentration in evacuated blood collection tubes evaluated with UV molecular absorption spectrometry on a purified water model. Molecules. (2023) 28:486. doi: 10.3390/molecules28020486, PMID: 36677544 PMC9860671

[16] Lin XC Zou HJ Wu X. The impact factors of evacuated blood collection on clinical laboratory assays. Chinese J. Infection Control. (2011) 3:235–7. (in Chinese). doi: 10.3969/j.issn.1671-9638.2011.03.023

[17] Najat D. Prevalence of pre-analytical errors in clinical chemistry diagnostic labs in Sulaimani City of Iraqi Kurdistan. PLoS One. (2017) 12:e0170211. doi: 10.1371/journal.pone.0170211, PMID: 28107395 PMC5249186

[18] Zemlin AE. Errors in the extra-analytical phases of clinical chemistry laboratory testing. Indian J Clin Biochem. (2018) 33:154–62. doi: 10.1007/s12291-017-0657-2, PMID: 29651205 PMC5891449

[19] Kang F Li W Xia X Shan Z. Three years’ experience of quality monitoring program on pre-analytical errors in China. J Clin Lab Anal. (2021) 35:e23699. doi: 10.1002/jcla.23699, PMID: 33458892 PMC7958002

[20] Peng MT Zhou WB Gu XL Li CB Wu J Lu H. WS/T 406–2012, analytical quality specifications for routine tests in clinical hematology. (2012). (in Chinese).

[21] Shanghai Clinical Laboratory Center, standard for quality control of clinical laboratories in Shanghai medical institution. (2019). (in Chinese)

